# Alterations in basal ganglia-cerebello-thalamo-cortical connectivity and whole brain functional network topology in Tourette's syndrome

**DOI:** 10.1016/j.nicl.2019.101998

**Published:** 2019-09-03

**Authors:** Shukti Ramkiran, Larissa Heidemeyer, Arnim Gaebler, N. Jon Shah, Irene Neuner

**Affiliations:** a Institute of Neuroscience and Medicine 4 (INM-4), Forschungszentrum Juelich, Juelich, Germany; b Department of Psychiatry, Psychotherapy and Psychosomatics, RWTH Aachen University, Aachen, Germany; c JARA – BRAIN – Translational Medicine, Germany; d Department of Neurology, RWTH Aachen University, Aachen, Germany; e Institute of Neuroscience and Medicine 11 (INM-11), JARA, Forschungszentrum Juelich, Juelich, Germany

**Keywords:** Tourette's syndrome, Connectivity, Basal ganglia, Cerebellum, Brain network topology

## Abstract

Tourette Syndrome (TS) is a neuropsychiatric disorder characterized by the presence of motor and vocal tics. Major pathophysiological theories posit a dysfunction of the cortico-striato-thalamo-cortical circuits as being a representative hallmark of the disease. Recent evidence suggests a more widespread dysfunction of brain networks in TS including the cerebellum and going even beyond classic motor pathways.

In order to characterize brain network dysfunction in TS, in this study we investigated functional and effective-like connectivity as well as topological changes of basal ganglia-thalamo-cortical and cortico-cerebellar brain networks. We collected resting-state fMRI data from 28 TS patients (age: 32 ± 11 years) and 28 age-matched, healthy controls (age: 31 ± 9 years). Region of interest based (ROI-ROI) bivariate correlation and ROI-ROI bivariate regression were employed as measures of functional and effective-like connectivity, respectively. Graph theoretical measures of centrality (degree, cost, betweenness centrality), functional segregation (clustering coefficient, local efficiency) and functional integration (average path length, global efficiency) were used to assess topological brain network changes.

In this study, TS patients exhibited increased basal ganglia-cortical and thalamo-cortical connectivity, reduced cortico-cerebellar connectivity, and an increase in parallel communication through the basal ganglia, thalamus and cerebellum (increased global efficiency). Additionally, we observed a reduction in serial information transfer (reduction in average path length) within the default mode and the salience network.

In summary, our findings show that TS is characterized by increased connectivity and functional integration of multiple basal ganglia-thalamo-cortical circuits, suggesting a predominance of excitatory neurotransmission and a lack of brain maturation. Moreover, topological changes of cortico-cerebellar and brain networks involved in interoception may be underestimated neural correlates of tics and the crucial premonitory urge feeling.

## Highlights

• Cerebellum shows reduced connectivity with the cortex in Tourette's syndrome (TS).
• Basal ganglia and thalamus show increased connectivity with the cortex in TS.
• Topological average path length is reduced in several cortical regions in TS.
• Topological efficiency is increased in the basal ganglia in TS.

## 1 Introduction

Tourette Syndrome (TS) is a neuropsychiatric disorder characterized by multiple motor and at least one or more vocal/phonic tics (American Psychiatric Association, 2000; Singer, 2005; Neuner and Ludolph, 2011). The clinical picture encompasses a complex, chronic and often disabling neuropsychiatric disease, which is often accompanied by several comorbidities, such as attention deficit disorder or obsessive-compulsive disorder (Cavanna et al., 2009).

There is a growing tendency to understand complex neuropsychiatric disorders such as TS as a ‘network problem’, and current studies have begun to asses a disturbed interplay within and between large-scale brain networks, rather than a localized dysfunction of specific single brain regions. As TS presents with many different complex manifestations of tics as well as many symptoms beyond tics such as comorbidities, this approach may be of particular interest.

A network of frontal areas, along with the basal ganglia, insula and cerebellum, have been highlighted as being significant in TS, suggesting that the basal ganglia-cerebellar-thalamo-cortical system plays a crucial role in the pathophysiology of tics (Singer, 2005; Leckman, 2002; Caligiore et al., 2017). In particular, tics are seen as a focal excitatory abnormality in the striatum, causing increased inhibition of the globus pallidus internus, leading to a disinhibition of of thalamic and - in turn -cortical neurons and therefore causing tics (Caligiore et al., 2017; Mink, 2001). More recently, several studies have shown abnormal activity in the cerebellum – a further key structure of the extrapyramidal system - during tic expression (Caligiore et al., 2017; McCairn et al., 2013; Neuner et al., 2014; Felling and Singer, 2011). This aberrant activity is hypothesized to be mediated by the disynaptic link from the basal ganglia to the cerebellum (McCairn et al., 2013), but this dysfunction still needs to be characterized in more detail.

Alterations in structural and functional connectivity have been suggested as key contributors to the psychopathology of TS (Neuner et al., 2014; Neuner et al., 2010). Using functional magnetic resonance imaging (fMRI), functional connectivity is commonly assessed during resting state, i.e. when the patient has no particular task to perform. Resting state-fMRI thus offers the advantage of a functional brain scan which is independent of the patients' cognitive capabilities, their cooperation or motivation. Moreover, since tics are discrete and dominate in the absence of other stimuli to focus attention on(Misirlisoy et al., 2015*)*, resting state represents a brain state which is related to the occurrence of tics and rendering it therefore more likely to detect abnormalities in TS patients. Abnormalities in resting state networks (networks of distributed brain regions exhibiting synchronized spontaneous fluctuations of the Blood oxygen level dependent (BOLD) signal) have been detected in different neurological (Boly et al., 2009; Werner et al., 2014) as well as psychiatric disorders (Bluhm et al., 2007; Sripada et al., 2012) including TS (Neuner et al., 2014; Cui et al., 2014; Worbe et al., 2012).

Network topological assessment of brain networks derived from connectivity data enables a better understanding of communication strategies employed by the brain. Key regions (network hubs) that function as information integration centres can be identified through centrality measures such as degree, betweenness centrality and cost. The values of these measures, in turn, reflect the importance of a particular node in a network. Other measures, such as average path length, global efficiency, local efficiency and clustering coefficient, provide information on the integration ability or the segregation ability of the node(Stam, 2019; Achard and Bullmore, 2007; Rubinov and Sporns, 2010). Previous studies have shown disruptions in the balance between local specialization and global integration mechanisms in whole brain-structural networks (Wen et al., 2017), and defects in network maturation, reflected by losses of hub regions in resting state cortico-basal ganglia functional networks (Worbe et al., 2012) in TS.

Given the importance of resting state and evidence on the involvement of the basal ganglia-cerebellar-thalamo-cortical system in TS, in this study, we focussed on resting state fMRI connectivity in TS with the aim of answering the following questions:

▪ Do the basal ganglia, the thalamus and the cerebellum exhibit different functional connectivity patterns with the cortex in TS patients at rest? We expected increased and widespread communication of the basal ganglia and thalamus with the cortex, which should be reflected by increased functional and effective-like connectivity. Furthermore, we intended to determine the nature of cortico-cerebellar dysconnectivity in TS.
▪ If the functional connectivity in TS patients looks different, then does the network organization of the brain look different as well? We expected an increase in functional integration mechanisms in the basal ganglia and thalamus, supporting the increase in connectivity, and further aimed to identify specific cortical and subcortical regions involved in the pathophysiology of tic disorders.

## 2 Materials and methods

### 2.1 Participants

28 adult out-patients (age: 32 ± 11 years) fulfilling the DSM-IV-TR criteria for TS and 28 age-matched, healthy controls (age: 31 ± 9 years) took part in this study. In the TS group, five patients additionally suffered from obsessive-compulsive disorder (OCD), and two from attention-deficit-hyperactivity disorder (ADHD), according to DSM-IV guidelines. Fourteen of the patients were receiving medication. Healthy controls were recruited from RWTH Aachen University and Forschungszentrum Juelich. Subsets of subjects from this dataset with different analyses focuses have been previously published in (Neuner et al., 2014; Neuner et al., 2012). The demographic details of the patients and controls can be found in supplementary Table S1, Table S2, respectively.

### 2.2 Data acquisition

Resting-state fMRI data were recorded with a 1.5 T MR system (Sonata, Siemens, Germany), using an echo-planar imaging protocol with the following parameters: T2∗-weighted echo-planar images, TE = 60 ms, TR = 3200 ms, 30 slices, 10% gap, FOV = 200 mm, in-plane resolution = 3.125 × 3.125 mm. Subjects were asked to keep their eyes closed without sleeping, and 220 volumes were acquired continuously for a total duration of 12 min. Structural images were acquired using a T1-weighted, 3D gradient-echo pulse sequence (MP-RAGE, magnetisation-prepared, rapid acquisition gradient echo) with the following parameters: TI = 1200 ms, TR = 2200 ms, TE = 3.93 ms, 15°flip angle, FOV = 256 × 256 mm^2^, matrix size = 256 × 256, 176 sagittal slices generated, slice thickness = 1 mm, resolution = 1 mm isotropic (voxel size = 1 mm^3^). An MR-compatible video camera system was used to monitor tics in the TS patients (Neuner et al., 2007).

### 2.3 Data preprocessing

The fMRI and sMRI data were processed using CONN v17.c (Whitfield-Gabrieli and Nieto-Castanon, 2012), a functional connectivity toolbox based on SPM12. Pre-processing of the structural MRI images involved the translation of the image centre (to the origin (0,0,0)), and unified segmentation and MNI- normalization. The unified segmentation model combines segmentation, registration and MNI-normalization in one model (Ashburner and Friston, 2005). Pre-processing of the functional MRI images involved the realignment and unwarp (for head motion correction), the translation of the image centre (to the origin (0,0,0)), slice-time correction, ART based outlier scan detection and scrubbing (for removal of volumes with excessive head-motion), and the MNI-space normalization of functional MRI images. Preprocessing was followed by nuisance regression of the functional MRI images. These noise variables were the 6 realignment parameters and their first derivatives, the scrubbing vectors, and the first 5 principal components of the times series' of WM and CSF (CompCor (Whitfield-Gabrieli and Nieto-Castanon, 2012)). This was followed by temporal band-pass filtering at 0.008–0.09 Hz and linear detrending.

### 2.4 Data analysis

#### 2.4.1 Connectivity

Following cleaning, ROI-ROI functional connectivity (FUN) and effective-like connectivity (EFF) analyses were performed using first-level analysis in CONN v17.c. Regions of interest (ROIs) were defined by the default atlas provided with CONN, which included 91 cortical and 15 subcortical parcellations from the Harvard-Oxford Maximum Likelihood atlas and 26 cerebellar parcellations from the AAL atlas. FUN analysis was performed by specifying a bivariate correlation model between ROIs and EFF analysis was performed by specifying a bivariate regression model between ROIs. Since our hypothesis specifically focussed on investigating the basal ganglia, thalamus and the cerebellum, at this point we investigated the connectivity of these 3 regions with all other ROIs. As per the atlas used, the basal ganglia included 8 ROIs, the thalamus 2 ROIs and the cerebellum 26 ROIs. All correlation and regression coefficient values were transformed using Fisher's z-transformation (hyperbolic arctangent). The connectivity differences between the TS patients and healthy volunteers was estimated by specifying a group contrast of [1–1] in the second level analysis of CONN, which performed a *t*-test on the connections and retained only the significant connections. Statistical analyses are explained in detail under section 2.5.

#### 2.4.2 Graph theory

Graph theory was applied to the obtained connectivity matrices after thresholding (> 0.4 or < −0.4) and binarizing them in order to investigate topological changes in the brain networks. This threshold value was chosen so that the resulting graph would only consist of the strong connections. Based on a previously unpublished study conducted in-house, we found the value of 0.4 optimal to study network topological properties and a good balance between a stringent and lenient threshold. The graph metrics we used can be divided into three categories as per the definitions by Rubinov and Sporns(Rubinov and Sporns, 2010).

##### 2.4.2.1 Measures of centrality

a) Degree – is the direct number of links to a node i.e. the number of node neighbours.
b) Cost – is the proportion of node neighbours with respect to the total number of nodes in the graph.
c) Betweenness centrality – is the ratio of short paths passing through a node to the total number of short paths in the graph. A short path is the minimum number of links that need to be passed when going from one node to the other.

These measures typically reflect the importance of a node in the entire network. A node with a high wiring cost and high betweenness centrality could be a hub integrating two small subnetworks, and dysfunction of this hub could lead to a dysfunction of both the subnetworks. A high degree reflects a high direct communication of a node with other nodes(Rubinov and Sporns, 2010).

##### 2.4.2.2 Measures of integration

a) Average path length – The average of short paths between a node and all other nodes is the average path length of that node.
b) Global efficiency – The average of all inverse short paths between this node and other nodes is defined as global efficiency.

These measures reflect how information between distinct brain regions and subnetworks is exchanged. They are typically path length-based measures(Rubinov and Sporns, 2010). While path length reflects the speed of serial information transfer through the node, global efficiency yields the speed of parallel information transfer(Achard and Bullmore, 2007).

##### 2.4.2.3 Measures of segregation

a) Local efficiency – is the average global efficiency of the neighbours of a node, excluding the node itself.
b) Clustering coefficient – is the proportion of a node's neighbours which are also connected to each other.

Typically, these are measures computed on the local subgraph of a node. The measures of local efficiency and clustering coefficient reflect information segregation ability of a node. In the case of functional brain networks, highly segregated networks or clusters of nodes could be specialized subnetworks responsible for a specific task. While clustering coefficient reflects the density and intra-connectedness of the subnetwork itself, local efficiency reflects the robustness of the subnetwork in the absence of the node(Latora and Marchiori, 2001).

### 2.5 Statistical analysis

The group comparison of connectivity differences was performed using a two-sample *t*-test. In order to correct for multiple comparisons, *p*-values were corrected for false discovery rates, and, finally, only those connections with a p-FDR corrected value < 0.05 were retained as significant. Each of the network metrics was also compared using a two-sample t-test and corrected for multiple comparisons using FDR correction. The regions showing alterations with a p-FDR < 0.05 were retained as significantly different.

**Fig. 1:**
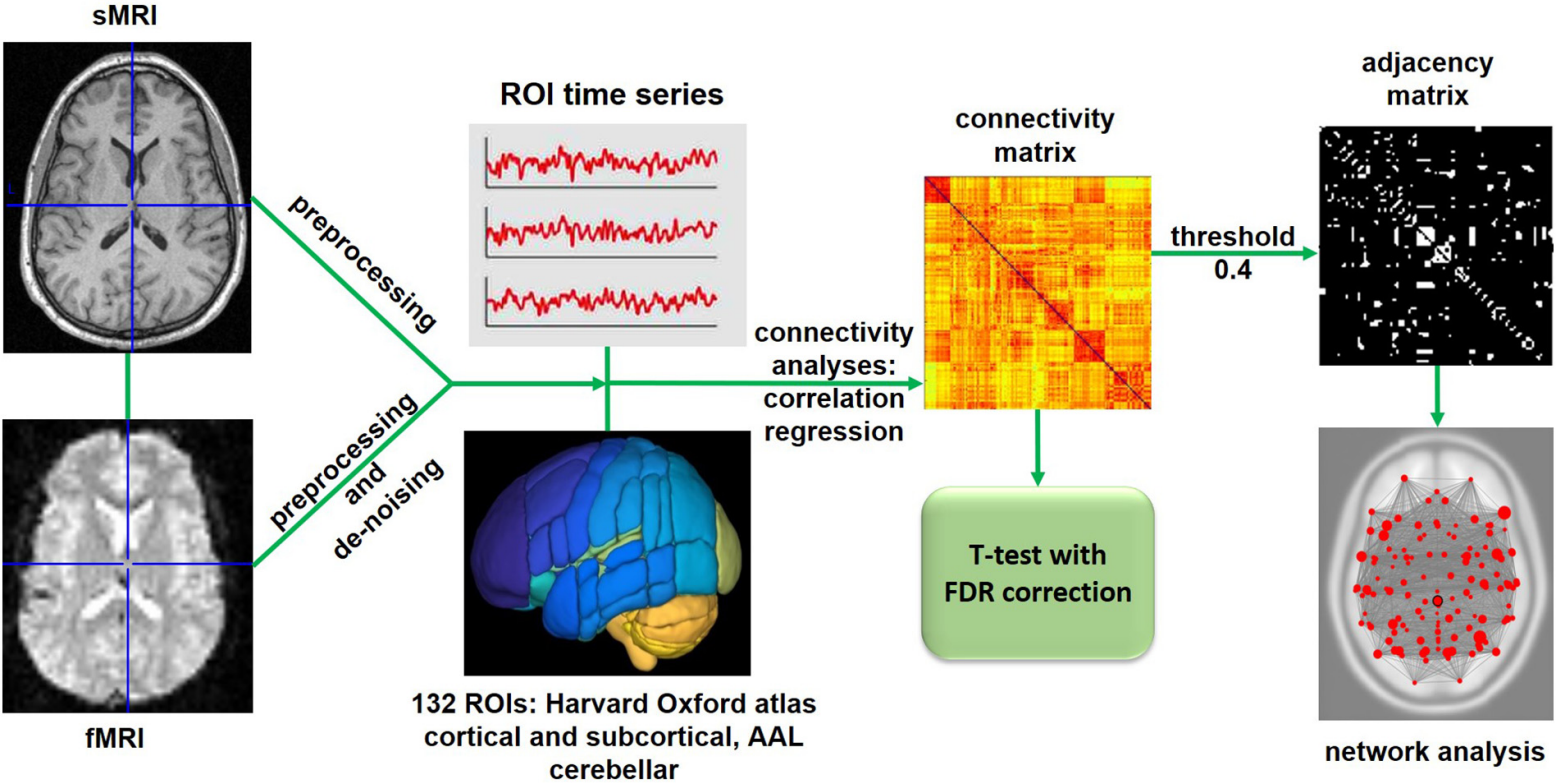
Data processing details (using toolbox CONN v17.c) – pre-processing of structural and functional images, denoising of functional images using GLM and band pass filtering, ROI-ROI bivariate correlation (functional connectivity – FUN) and regression (effective-like connectivity – EFF) analysis, network analysis using graph theory.

A flowchart of data processing can be seen in Fig. 1.

## 3 Results

**Fig. 2:**
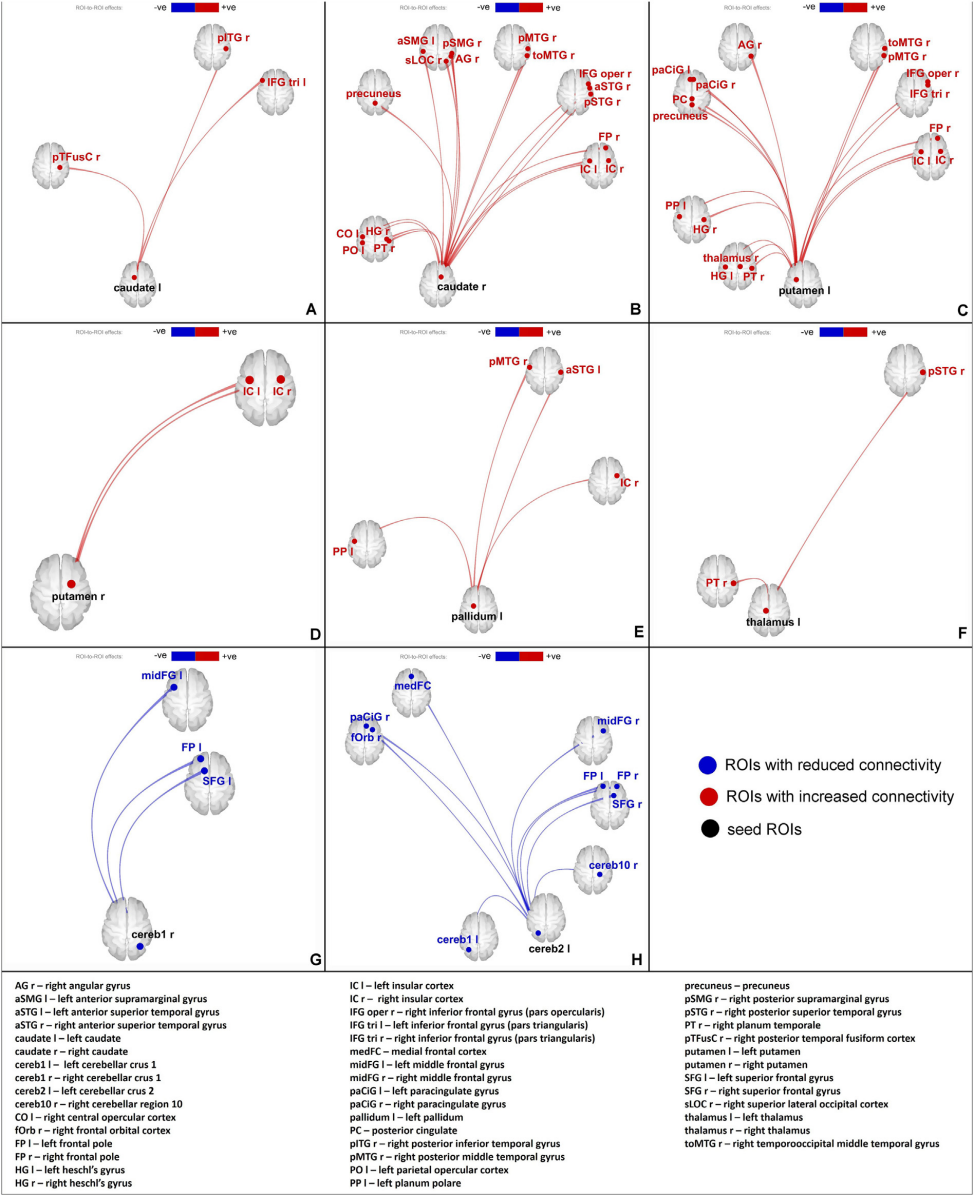
FUN alterations: increased connectivity of left caudate **(A)**, right caudate **(B)**, left putamen **(C)**, right putamen **(D)**, left pallidum **(E)** and left thalamus **(F)** with several other regions; reduced connectivity of right cerebellar crus 1 **(G)** and left cerebellar crus 2 **(H)** with other brain regions. Red – increased connectivity, Blue – reduced connectivity. (Connectome ring in supplementary Fig. S3). FUN alterations: increased connectivity of left caudate **(A)**, right caudate **(B)**, left putamen **(C)**, right putamen **(D)**, left pallidum **(E)** and left thalamus **(F)** with several other regions; reduced connectivity of right cerebellar crus 1 **(G)** and left cerebellar crus 2 **(H)** with other brain regions. Red – increased connectivity, Blue – reduced connectivity. (Connectome ring in supplementary Fig. S3).

**Fig. 3:**
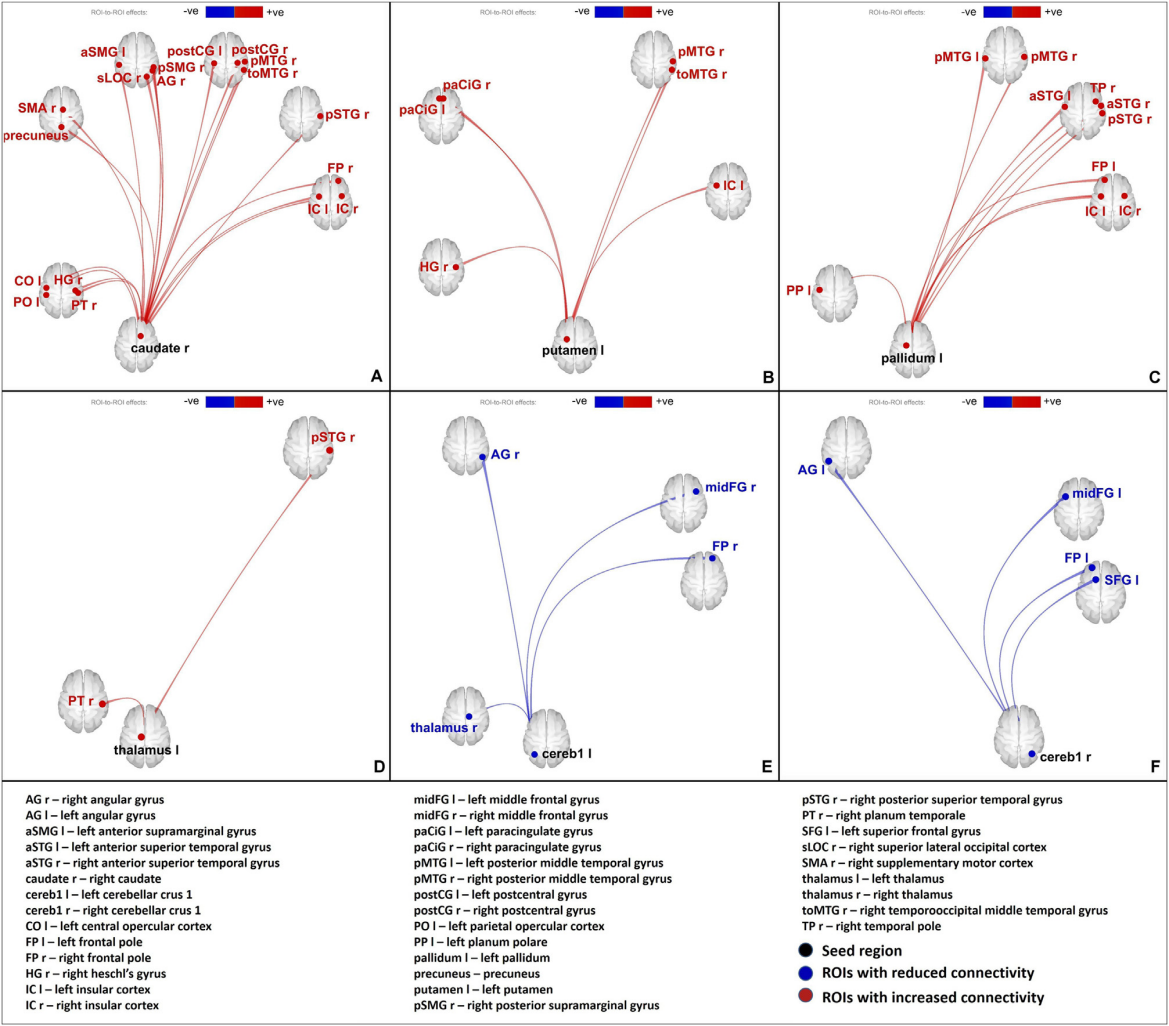
EFF alterations: increased effect of right caudate **(A)**, left putamen **(B)**, left pallidum **(C)** and left thalamus **(D)** on several other regions; reduced effect of left cerebellar crus 1 **(E)** and right cerebellar crus 1 **(H)** on other brain regions. Red – increased connectivity, Blue – reduced connectivity. (Connectome ring representation in supplementary Fig. S4). EFF alterations: increased effect of right caudate **(A)**, left putamen **(B)**, left pallidum **(C)** and left thalamus **(D)** on several other regions; reduced effect of left cerebellar crus 1 **(E)** and right cerebellar crus 1 **(H)** on other brain regions. Red – increased connectivity, Blue – reduced connectivity. (Connectome ring representation in supplementary Fig. S4).

**Fig. 4:**
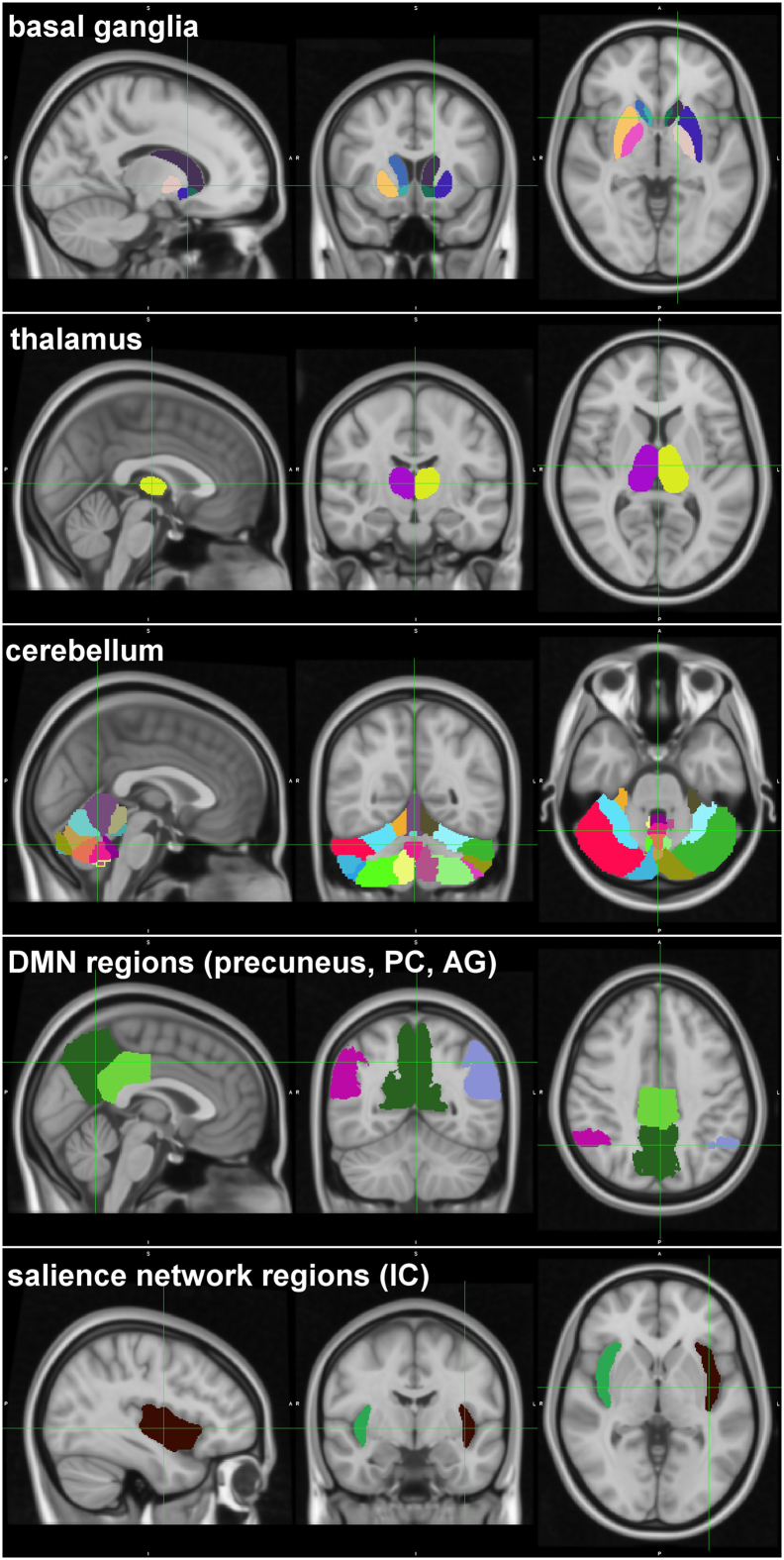
Parcellations of the different regions of interest we investigated – the basal ganglia (8 ROIs), thalamus (2 ROIs), cerebellum (26 ROIs), default mode network (4 regions), salience network (2 regions). Since the DMN and salience network were not specifically investigated, only those regions which showed alterations in TS in this study have been depicted here. The colour scheme is random, the different colours represent the different regions.

**Fig. 5:**
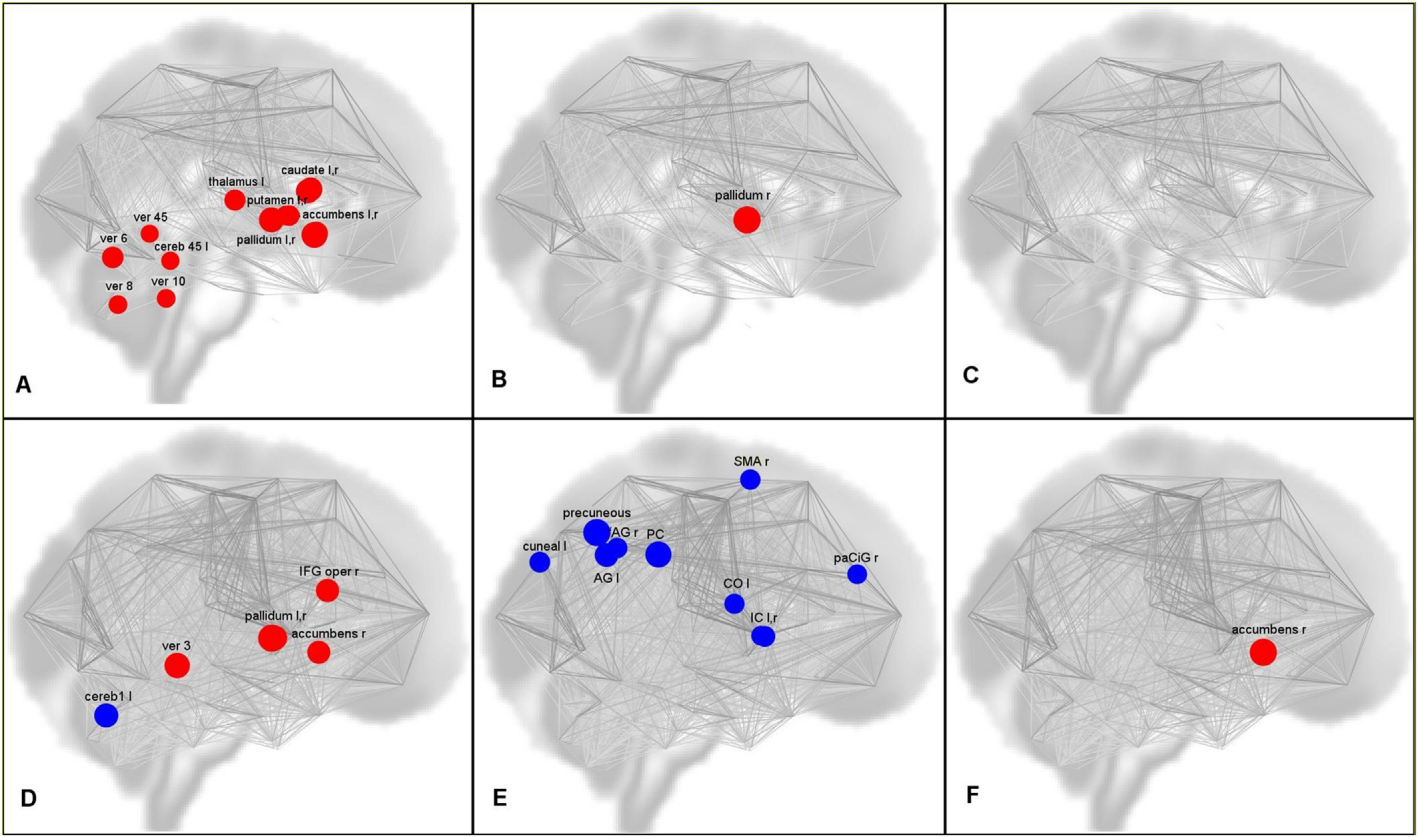
Network topological alterations observed in TS patients against healthy volunteers (contrast patients>controls) – FUN global efficiency (**A**), FUN average path length **(B)**, FUN clustering coefficient **(C)**, EFF global efficiency **(D)**, EFF average path length **(E)**, EFF clustering coefficient **(F).** Red – increased in TS patients, Blue – reduced in TS patients.

Significant differences were found in FUN and EFF connectivity between the TS patients and the healthy controls. Network analyses showed significant differences in the metrics global efficiency, average path length and clustering coefficient in either FUN or EFF networks. However, there were no alterations in measures of node importance, such as cost, degree and betweenness centrality, or in the measure reflecting the performance of neighbours i.e. the local efficiency, in either the FUN or the EFF network. Fig. 2, Fig. 3 show the connectivity differences, Fig. 4 shows the regions of interest we investigated and Fig. 5 reflects the network changes we observed. Specific changes observed in the different brain regions and networks are explained below.

### 3.1 Basal ganglia and thalamus

Increased FUN was observed between the basal ganglia nuclei (left and right caudate, putamen, left pallidum) and several regions of the cortex including (but not limited to) the superior temporal gyrus, medial temporal gyrus, paracingulate gyrus, precuneus, angular gyrus and insular cortex in TS patients when compared to healthy controls. EFF revealed similar results, however, the left caudate and right putamen did not show any significant connectivity alterations. Interhemispheric differences could be seen in the FUN results, as the number of altered connections from each of the nuclei differed between the left and right hemispheres. These differences are more prominent in the EFF results.

Both FUN and EFF analyses showed increased connectivity between the left thalamus and the cortex (right planum temporale, right superior temporal gyrus) in TS patients when compared to healthy controls.

Network analysis showed an increase in global efficiency in the left and right caudate, putamen, accumbens, pallidum and the left thalamus, and an increase in average path length in the right pallidum in the FUN network. In the EFF network, an increase in global efficiency in the right accumbens, left and right pallidum, and an increase in clustering coefficient in the right accumbens was observed.

### 3.2 Cerebellum

Reduction in FUN was observed between the cerebellum (subregions: right cerebellar parcellation 1, left cerebellar parcellation 2) and several cortical regions including (but not limited to) the superior frontal gyrus, middle frontal gyrus, frontal pole in TS patients when compared to healthy controls. Similar to the basal ganglia, the results from EFF were similar to the results from FUN in terms of cortico-cerebellar connectivity. Visual inspection of our data reflected that a greater number of patients showed negative connectivity in these cortico-cerebellar connections than healthy controls.

Network analyses showed an increase in global efficiency (subregions: cerebellar parcellation 3 and 4, cerebellar vermis parcellations 12, 45, 6, 7, 8) in the FUN network. The EEF network showed an increase in global efficiency in one subregion (cerebellar vermis parcellation 3) and a reduction in global efficiency in another subregion (left cerebellar crus parcellation 1).

### 3.3 Resting state networks

Although the connectivity analyses were focused only on the basal ganglia, the thalamus and the cerebellum, the whole brain network analyses additionally showed alterations in regions belonging to different resting state networks. The EFF network showed a decrease in average path length in several regions of the default mode network (precuneus, posterior cingulate, left and right angular gyrus) and the salience network (left and right insular cortex) in TS patients when compared to healthy controls. The FUN network, however, did not show any changes in regions involved in any of the resting state networks.

## 4 Discussion

Applying different measures of functional connectivity to resting-state fMRI data showed different brain function in TS patients compared to healthy controls. More precisely, we found increased functional and effective like connectivity of the basal ganglia and thalamus with the cortex. Through network analyses, we primarily found alterations in functional information integration mechanisms, which were reflected by global efficiency and average path length; an exception being in the accumbens, which also showed alterations in functional segregation reflected by the clustering coefficient. However, the importance of the regions remained unaltered, as reflected by the centrality measures. Based on this, we can argue that while the overall network structure remained preserved, information exchange mechanisms differed in patients with TS. Our findings extend major pathophysiological theories by suggesting a pivotal role of cortico-cerebellar circuits in addition to basal ganglia-thalamo-cortical networks.

### 4.1 Basal ganglia and thalamus

As a major finding, TS patients exhibited increased functional connectivity between the basal ganglia, the thalamus and multiple cortical areas. As a complementary finding, both the basal ganglia and the thalamus exhibited increased functional integration, reflected by the increase in average path length and global efficiency in TS patients. In classic pathophysiological models, hyperkinetic disorders originate from dysfunctional basal ganglia -thalamo-cortical circuits, leading to a hyper-activation of the motor cortex. Within this framework, tics are thought to result from abnormal excitation of the striatum leading to increased inhibition of neurons in the globus pallidus internus and in turn a disinhibition of thalamic and cortical neurons. Accordingly, our findings indicate excessive communication between the basal ganglia and the cortex. As a neurophysiological correlate, one would expect a general increase of glutamatergic or a decrease of GABAergic signalling. Indeed, previous spectroscopy studies have confirmed increased glutamate levels in the striatum and premotor cortex. Additionally, PET studies have revealed the reduced binding potential of 11-C-flumazenil (a GABAergic radiotracer) in the basal ganglia and thalamic regions in TS patients (Felling and Singer, 2011) and post mortem studies have demonstrated a loss of striatal GABAergic inhibitory interneurons(Robertson et al., 2017).

In accordance with our findings, Worbe et al. demonstrated increased global functional integration of different cortico-basal ganglia networks in TS patients which was interpreted as indicating deficient brain maturation and thus supporting the neurodevelopmental hypothesis of TS (Worbe et al., 2012).

Interestingly, hyperconnectivity of the basal ganglia and thalamus included several cortical areas exceeding classic motor pathways. Many of these areas have previously been implicated in the pathophysiology of tics (explained further in section 4.3 below).

#### 4.1.1 Nucleus accumbens

Interestingly, the nucleus accumbens did not show any connectivity changes but instead showed changes in functional segregation as reflected by the increase in clustering coefficient in the EFF network. This finding might be explained by the fact that – in contrast to the dorsal striatum - the ventral striatum (accumbens) receives glutamatergic inputs from the limbic system rather than the cortex. Its increased clustering coefficient points to the presence of a more densely connected subnetwork to achieve its task in TS patients as compared to healthy volunteers. Our findings are supported by evidence of reduced FDG-PET metabolic activity(Braun et al., 1993), which shows that the accumbens is using less energy to perform its tasks in TS patients than in healthy volunteers. Further evidence for its pathophysiological importance comes from deep brain stimulation studies, which have revealed that stimulation of the accumbens leads to substantial tic reduction(Neuner et al., 2014).

### 4.2 Cerebellum

The cerebellum represents a major neural substrate of motor and language control. However, it is still underrepresented in TS research and -whereas growing evidence suggests its affection in TS patients-, cerebellar dysfunction in TS still has to be characterized in detail. Cerebellar activation has been consistently observed during tic performance in patients with TS (Caligiore et al., 2017; Neuner et al., 2014) and animal models (McCairn et al., 2013). Moreover, Tobe et al. identified bilateral volume reductions of the cerebellum in TS patients which correlated with tic severity and motoric disinhibition (Tobe et al., 2010).

As an anatomical basis, the cerebellum exhibits reciprocal connections both with the cortex and the basal ganglia. A recent computational model (Caligiore et al., 2017) suggests that the cerebellum is indeed involved in tic production mediated by the recently discovered subthalamic-pons-cerebellar circuit.

In our study, we detected an increased parallel information transfer through the cerebellar regions as indicated by the increase in global efficiency. This finding, together with our finding on reduced connectivity, could be indicative of an increase in negative connectivity (anticorrelations) between the cerebellar and the cortical regions. Indeed, our data reflected that a greater number of patients showed negative connectivity in these cortico-cerebellar connections than healthy controls. Anticorrelations reflect either inhibitory activity or functional differentiation (Ramkiran et al., 2019). Increased inhibitory signalling mediated by cortico-cerebellar projections is also supported by evidence from PET studies showing an increased binding potential of the GABAergic tracer 11C-Flumazenil in the cerebellum (Felling and Singer, 2011). We suppose that increased inhibitory activity mediated by cortico-cerebellar projections might reflect a compensatory mechanism to supress the performance of tics. Future studies are warranted to further clarify this finding.

### 4.3 Resting state networks: salience network (insula) and default mode network (precuneus, posterior cingulate and angular gyrus)

In TS patients, the right putamen and the left pallidum showed increased functional connectivity with the bilateral insular cortex. Moreover, the insular cortex exhibited a reduction in average path length, indicating a decrease in serial information transfer through the insula. Indeed, the insula has been shown to be active before - but not during- tic onset (Neuner et al., 2014).

The insula and anterior cingulate cortex constitute the salience network which has increasingly become the focus of attention for its role in body representation, interoception, food and drug craving, salience detection and subjective emotional experience (Lamm and Singer, 2010; Phan et al., 2002; Mutschler et al., 2007; Xue et al., 2010). Given its involvement in craving and body representation, increased functional connectivity between the insula and basal ganglia may be a neural correlate of the premonitory urge feeling which typically precedes the onset of tics in TS patients. In accordance with this interpretation, Tinaz and colleagues (Tinaz et al., 2015) suggest that the right dorsal anterior insula is part of the urge-tic network, as it was correlated positively with the tic severity in patients. Therefore, it was suggested that the right dorsal anterior insula might potentially be influencing the urge- and tic-related cortico-striato-thalamic regions even during rest in TS. A further neural substrate of the premonitory urge feeling might be the default mode network comprising the medial prefrontal gyrus, posterior cingulate, precuneus and the bilateral angular gyrus.

Indeed, our data revealed that the right caudate, as well as the right thalamus and left putamen, exhibited increased functional connectivity with the precuneus. Moreover, network analysis showed a decrease in average path length in the precuneus, posterior cingulate and the angular gyrus, indicating a decrease in serial information transfer through these regions. The default mode network is particularly involved in processes related to self-awareness such as self- reference and autobiographic memory retrieval (Cavanna and Trimble, 2006). Increased connectivity between the basal ganglia and the default mode network may, thus, represent a neural correlate of self-referential thinking related to tics, e.g. in the context of the premonitory urge feeling (Ganos et al., 2015). Therefore, we hypothesize that both the salience and default mode network are involved in the premonitory urge feeling. Recent evidence suggests that the salience and default mode network may be considered as a unified large-scale brain system supporting interoception and allostasis (Kleckner et al., 2017), highlighting our premonitory urge hypothesis.

## 5 Limitations of the study

The sample of TS participants is heterogeneous, since some patients were medicated and some not. Some of the patients also suffered from other comorbidities. Furthermore, doses and medications were not homogenous in the cohort. This might have influenced the results.

## 6 Conclusions

The present study demonstrated global and functional disorganization of the cortico-basal ganglia networks in TS. Functional and effective-like connectivity analyses revealed altered connectivity in the basal ganglia, thalamus and cerebellum. Network topological changes were most prominent in parts of the basal ganglia (motor-control), default mode network (self-awareness), insular cortex (craving, emotion and salience processing), nucleus accumbens (emotional distress triggering tics) and the cerebellum (coordination, precision, cognition). These results reflect a highly complex process leading to tic formation.

The following are the supplementary data related to this article.

Table S1 Demographic details of TS patients. Table S1

Table S2 Demographic details of healthy volunteers. Table S2

**Fig. S3:**
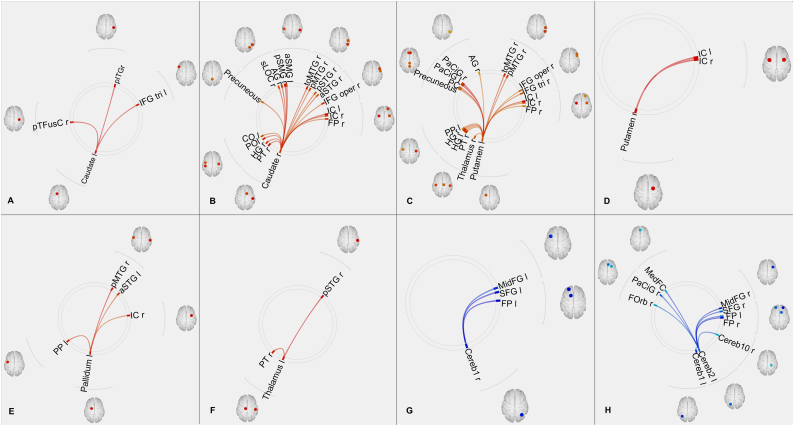
Connectome ring representation for differences in FUN between patients and healthy volunteers. Red - increased, blue - reduced in patients when compared to healthy volunteers.

**Fig. S4:**
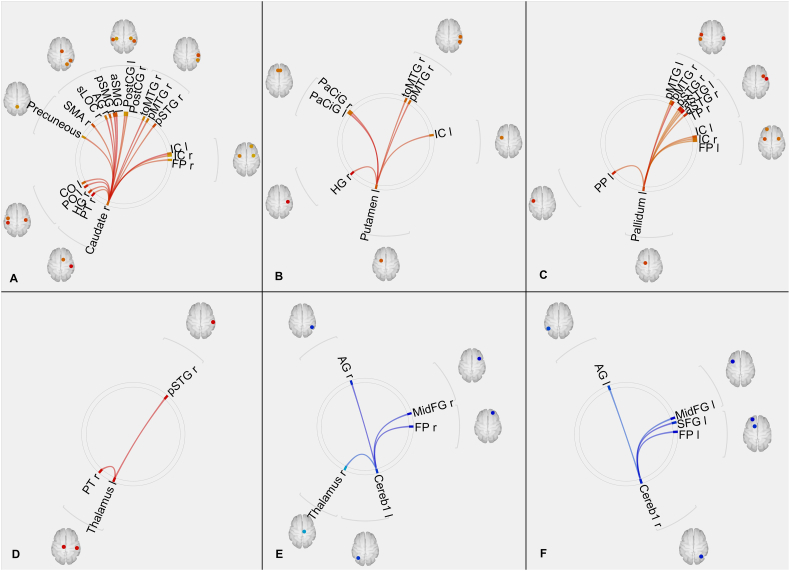
Connectome ring representation for differences in EFF between patients and healthy volunteers. Red - increased, blue - reduced in patients when compared to healthy volunteers.

## Declaration of Competing Interest

None.
