# Supplementary material for: Alterations in basal ganglia-cerebello-thalamo-cortical connectivity and whole brain functional network topology in Tourette's syndrome
Source: Neuroimage Clin. 2019 Sep 3;24:101998. doi: 10.1016/j.nicl.2019.101998 (PMC6742843; doi:10.1016/j.nicl.2019.101998)
Supplement: Table S1 — Demographic details of TS patients. [file mmc1.doc]

**Table S1** : demographic details of TS patients

| Patient | Age | Gender | Medication | YGTTS |
| --- | --- | --- | --- | --- |
| 1 | 41 | f | 0.00 | 33.00 |
| 2 | 22 | f | 0.00 | 57.00 |
| 3 | 56 | f | 40 CIT, 400 CBZ | 63 |
| 4 | 46 | f | 0.00 | 46.00 |
| 5 | 34 | f | 2 PIM | 47.00 |
| 6 | 29 | f | 20 CIT, 50 MPH | 53.00 |
| 7 | 32 | m | 100 TRIM | 67.00 |
| 8 | 36 | m | 0.00 | 68.00 |
| 9 | 26 | m | 10 ARI | 74.00 |
| 10 | 21 | m | 10 ESC | 40.00 |
| 11 | 45 | m | 200 TIA | 59.00 |
| 12 | 21 | m | 4 PIM incompliant | 77.00 |
| 13 | 49 | m | 0.00 | 37 |
| 14 | 46 | m | 0.00 | 54.00 |
| 15 | 28 | m | 10 ARI | 58.00 |
| 16 | 28 | m | 0.00 | 17.00 |
| 17 | 48 | m | 0.00 | 57.00 |
| 18 | 39 | m | 200 AMS, 50 TIA | 80.00 |
| 19 | 49 | m | 0.00 | 37.00 |
| 20 | 24 | m | 0.00 | 2.00 |
| 21 | 21 | m | 0.00 | 59.00 |
| 22 | 27 | m | 0.00 | 63.00 |
| 23 | 25 | m | 20 CIT, 200 TIA | 66.00 |
| 24 | 26 | m | 50 TRIM | 44.00 |
| 25 | 22 | f | 10 ARI, 20 FLX | 27.00 |
| 26 | 19 | m | 80 ZPR | 60.00 |
| 27 | 21 | m | 0.00 | 62.00 |
| 28 | 26 | m | 0.00 | 63 |

| CIT - CITALOPRAM |
| --- |
| CBZ - CARBAMAZEPINE |
| PIM - PIMOZIDE |
| MPH - METHYLPHENIDATE |
| TIA - TIAPRIDE |
| AMS - AMISULPIRIDE |

| TRIM - TRIMIPRAMINE |
| --- |
| FLX - FLUOXETINE |
| ZPR - ZIPRASIDONE |
| ARI - ARIPIPRAZOLE |
| ESC - ESCITALOPRAM |
