# Supplementary material for: Alterations in basal ganglia-cerebello-thalamo-cortical connectivity and whole brain functional network topology in Tourette's syndrome
Source: Neuroimage Clin. 2019 Sep 3;24:101998. doi: 10.1016/j.nicl.2019.101998 (PMC6742843; doi:10.1016/j.nicl.2019.101998)
Supplement: Table S2 — Demographic details of healthy volunteers. [file mmc2.doc]

**Table S2** : demographic details of healthy volunteers

| Control | Age | Gender |
| --- | --- | --- |
| 1 | 26 | f |
| 2 | 40 | f |
| 3 | 25 | f |
| 4 | 23 | f |
| 5 | 28 | f |
| 6 | 29 | f |
| 7 | 45 | f |
| 8 | 33 | f |
| 9 | 43 | f |
| 10 | 26 | m |
| 11 | 23 | m |
| 12 | 26 | m |
| 13 | 26 | m |
| 14 | 54 | m |
| 15 | 25 | m |
| 16 | 48 | m |
| 17 | 36 | m |
| 18 | 21 | m |
| 19 | 23 | m |
| 20 | 27 | m |
| 21 | 21 | m |
| 22 | 27 | m |
| 23 | 41 | m |
| 24 | 42 | m |
| 25 | 42 | m |
| 26 | 25 | m |
| 27 | 31 | m |
| 28 | 25 | m |
